# Vitamin D levels and parathyroid hormone variations of children living in a subtropical climate: a data mining study

**DOI:** 10.1186/s13052-018-0479-8

**Published:** 2018-03-21

**Authors:** Ozlem Naciye Sahin, Muhittin Serdar, Mustafa Serteser, Ibrahim Unsal, Aysel Ozpinar

**Affiliations:** 1 Department of Pediatrics, Acibadem Mehmet Ali Aydinlar University, School of Medicine, Icerenkoy mah. Kayısdagı cad. No.32, 34752 Istanbul, Atasehir Turkey; 2 Department of Clinical Biochemistry, Acibadem Mehmet Ali Aydinlar University, School of Medicine, Istanbul, Turkey

## Abstract

**Background:**

Vitamin D and intact parathyroid hormone (iPTH) play a crucial role in calcium homeostasis and bone health of children. Serum level of 25-hydroxyvitamin D (25-OHD) is considered to be the most accurate marker for vitamin D status. However, there have only been a few studies, with limited number of subjects, investigating the relationship between 25-OHD and parathyroid hormone (PTH) in children. The aim of this study was to evaluate the seasonal 25-OHD levels and its associations with intact parathyroid hormone (iPTH) in Turkish children at all pediatric ages; and then to define a critical decision threshold level for 25-OHD deficiency in Turkish children.

**Methods:**

A retrospective record review of 90,042 children, was performed on serum 25-OHD and for 3525 iPTH levels. They were measured by mass spectrometry method and by electrochemiluminescence immunoassay simultaneously.

**Results:**

25-OHD levels showed a sinusoidal fluctuation througout the year; being significantly higher in summer and autumn (*p* < 0,01). 25-OHD levels decreased with respect to age. The significant inverse relationship that was found between iPTH and 25-OHD suggests that the inflection point of serum 25-OHD level for maximal suppression of PTH is at 30 ng/ml.

**Conclusion:**

As the rate of vitamin D deficiency decreases in the early years due to vitamin D supplementation, the recommendation should be set due to a clinical threshold level of 30 ng/ml for 25-OHD based on PTH levels in children of our population.

## Background

Vitamin D is a prohormone synthesized in the skin after exposure to ultraviolet radiation. This prohormone is transformed into its metabolically active form in the liver and kidneys. Cholecalciferol (vitamin D3) is formed when UV-B (ultraviolet-B radiation) (wavelength 290-315 nm) converts 7-dehydrocholesterol in epidermal keratinocytes and dermal fibroblasts to vitamin D, which subsequently isomerizes to D3. In the kidney, 25- OHD undergoes 1 alfa hydroxylation to form 1,25 (OH)_2_, the active form of the vitamin, also designated as calcitriol. 25-OHD is the primary metabolite and is mainly used to evaluate vitamin D status [1]. Steps through the synthesis of active vitamin D is controlled by iPTH and some other mediators. Severe vitamin D deficiency causes rickets and/or hypocalcemia in infants and children; and osteomalacia in adolescents and adults. Rickets is due mainly to vitamin D deficiency, and was common in northern Europe and America during the early years of the twentieth century [2]. The diagnosis of Ricketts is based on radiological findings, physical examination, history and supported by laboratory tests. The clinical evaluation should focus on a dietary history, mainly on calcium and vitamin D levels. The IOM recommends that the best marker of vitamin D deficiency is a 25-OHD measurement [3]. It reflects mainly the endogenous vitamin D synthesis and vitamin D supplementation. Humans obtain > 80% of their vitamin D via exposure to the UV-B component of sunlight. There is also a significant variation in the circulating levels of 25-hydroxyvitamin D3 in nonequatorial regions. The normal vitamin D levels are considered to vary based on different biological needs but > 50 nmol L^−^ 1(> 30 ng/mL ^− 1^) is currently considered optimal, whilst 25-OHD between 20 and 30 ng/mL (37.5–50 nmol/L) L may be insufficient, and < 20 ng/mL (37.5 nmol/L) deficient [4]. Due to the seasonal, age and gender variations measurement of 25(OH)D levels, a diagnosis of vitamin D deficiency becomes methodologicaly challengeing [5–9]. In addition to these challenges, ethnic variations may present different thresholds for iPTH activation at different vitamin D cut off levels. The reference interval calculations cannot be performed by classical methods that causes a significant problem in methodology [10]. Assessment of Vitamin D status can be assayed using radioimmunoassays, competetive protein binding assays (CPBA), high pressure liquid chromatography (HPLC), and liquid chromatography-tandem mass spectrometry. Because of an inter-assay variation these methods may yield different results reaching up to 25% (at lower serum levels) and intra-assay variation reaching 10%. The optimal serum vitamin D concentration has not been established and may change across different stages of life. Therefore, no consensus has been reached which determines the clinical decision levels for Vitamin D [11, 12].

Data mining is the study of efficiently finding structures and patterns in large data sets. It converts raw messy data set to a structured form; applies scalable and probabalistic algorithms to the newly structured abstract data sets, and then models them. Remarkable biotechnological and health science advances has led to a significant production of data generation from large Electronic Health Records (EHR). To this end, the application of data mining methods to biosciences has more than ever before been able to transform raw available information into valuable knowledge [13]. Another valuable use of patient data lies in the arena of clinical and health services research. This has led to the identification of health effects from environmental factors through the discovery of patterns otherwise unknown [14]. Data mining, however, has some limitation; it may increase the number and the range of confounders in data analysis.

In this study we analyzed the changes in 25-OHD and PTH levels using the tandem mass spectrometry technique; currently accepted as the most stable method for this type of mesurements. Our retrospective data involves the largest data set, in terms of our pediatric population, currently available.

## Methods

A retrospective record review was carried out on children aged 2 months to 18 years. Our study included test results of 25-OHD and iPTH, from 90,042 pediatric samples. The anonymized and de-identified laboratory data of all pediatric patients that had 25-OHD and iPTH levels measured from 2010 to 2016 was retrieved from the laboratory database of Acibadem clinical laboratories (Turkey). Extreme values were excluded using Studentized procedure. This left 47,928 female and 42,114 male test results eligible for vitamin D, and 1798 female and 1727 male test results eligible for iPTH data analysis. Serum 25-OHD concentrations were measured using Agilent Rapid Res 1200 LC system and Agilent 6460 triple quadruple mass spectrometer (Agilent Technologies, Santa Clara, CA). iPTH concentrations were determined by an electrochemiluminescence immunoassay with Elecsys analyzer (Roche Diagnostics, Mannheim, Germany). In reference to iPTH, values greater than 200 pg/mL were excluded as they corresponded to a three-fold increase beyond the upper limit of normal.

Analyse-it for Microsoft Excel 4.0, and Medcalc 15.8 (Medcalc Company), were used for statistical analysis in this study. ANOVA and post-hoc Tukey analysis were used to compare age and month. Independent sample t test was used to compare genders. Regression analysis was applied for the relationship between PTH and 25-OHD. The significance level of *p* was defined as < 0.01. Procedures were all in accordance with the ethical standards set by the responsible human experimentation comittee (institutional and national), as well as the latest version of Declaration of Helsinki as given by World Medical Association. The ethical approval of this study was obtained from Muğla Sıtkı Kocman University Ethics comittee; protocol number 6(143).

## Results

**Fig. 1:**
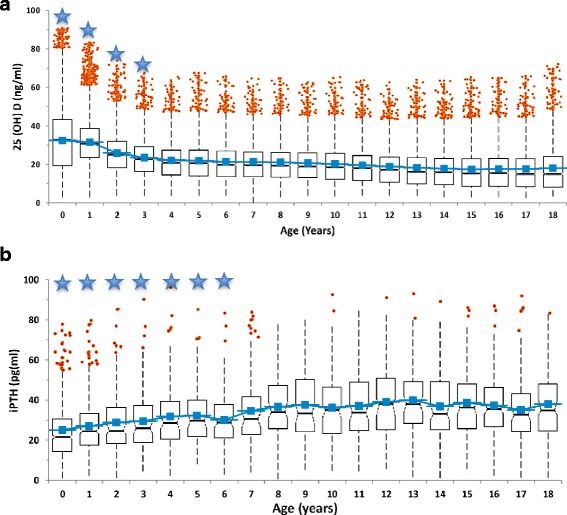
25-OHD levels (**a**) and iPTH levels (**b**) according to age. Star sign: there is a significant difference (*p* < 0.01)

**Fig. 2:**
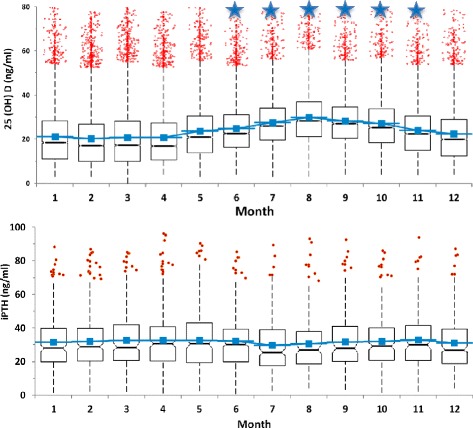
25-OHD and iPTH hormone levels according to months. Star sign: there is significant difference (*p* < 0.01)

**Fig. 3:**
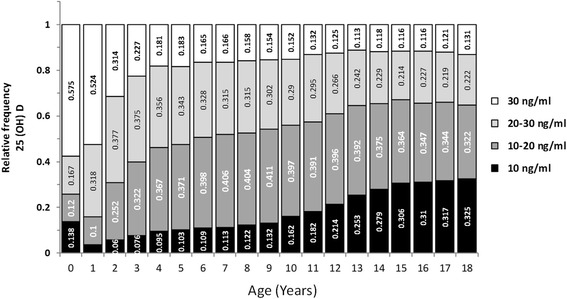
The prevalence of Vitamin D deficiency

**Fig. 4:**
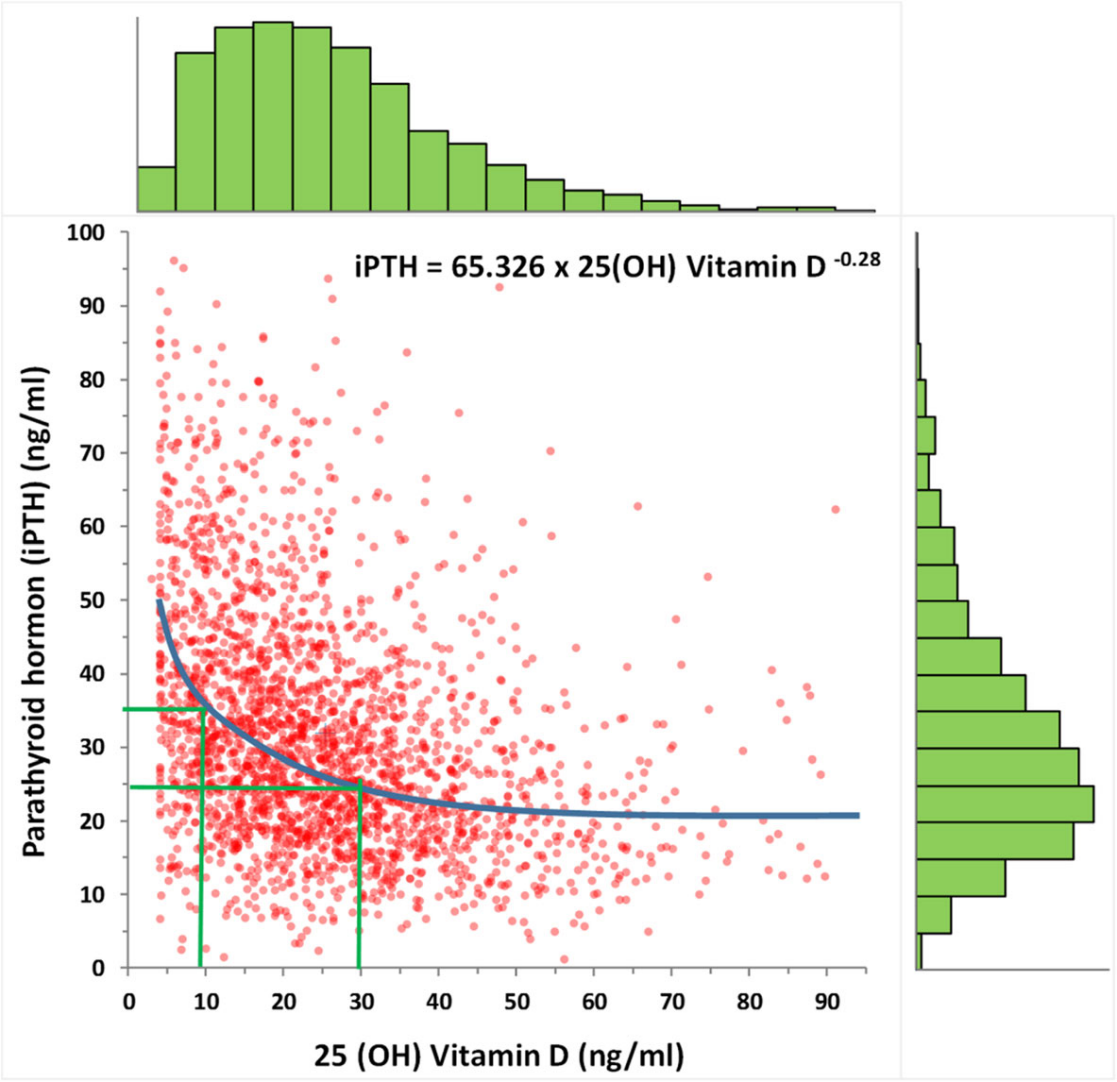
The inverse relationship between 25(OH)D and iPTH

25-OHD levels of 90,042 children and iPTH levels of 3535 children were measured and evaluated, as shown in Table 1, according to age and gender. Considering serum 25-OHD in preschool and school children, preschool children had significantly higher 25-OHD levels than those of school children. Accordingly, iPTH levels increased until age of 8, before seeming to plateu later (Fig. 1). Analysis of 25-OHD and iPTH hormone levels demonstrated a sinusoidal pattern, with a significant increase in June, a peak in August and a decrease to lower levels in December (Fig. 2.). There was a near two fold difference in median 25-OHD values between August and March levels. Considering the seasonal August and March sinusoidal pattern of vitamin D concentrations, iPTH showed an inverse sinusoidal pattern. Gender difference for 25-OHD and iPTH levels were statistically significant as shown in Table 1. Vitamin D levels were significantly lower in females, while females also had significantly increased iPTH levels. On the analysis of seasonal gender and age effects on vitamin D levels, children had low 25-OHD levels between February and May (Fig. 2). The population size with low 25-OHD levels was small during summer months. The prevalence of 25-OHD deficiency was seen to increase and become more prominant as the children grew to the age of 18 (Fig. 3). The deficiency is observed at 40–45% in early ages, before rising to as much as 80–90% after the age of 10. Here there was a significant inverse correlation between 25-OHD and iPTH levels (Fig. 4). This correlation was nonlinear and plateaued when iPTH levels were below 25 ng/mL. The relationship between 25-OHD and iPTH was exponential and statistically significant (iPTH = 65.326 × 25-OHD^-0.28^, *p* < 0.001). This finding is unique when compared to adults at every 10 years while in this study we searched for vitamin D versus iPTH relationship at all pediatric ages transition of the 25-OHD/iPTH relationship from linear to plateu cut-off point was considered as 30 ng/mL.

**Table 1:** 25-OHD levels and iPTH levels according to age and gender

	25 (OH)D (mg/ml)						iPTH(ng/ml)					
	Female			Male			Female			Male		
	n	Mean	SD	n	Mean	SD	n	Mean	SD	n	Mean	SD
0	6576	30,5	18,1	5959	34,7	16,5	296	24,1	15,4	434	25,7	15,1
1	6307	31,4	12,9	6707	31,8	12,6	212	26,9	13,8	234	27,4	13,0
2	2701	25,6	11,5	2974	26,2	11,2	105	30,0	16,8	122	27,7	14,2
3	2066	23,3	10,6	2499	23,7	10,7	81	30,9	14,2	77	28,0	16,2
4	1879	21,3	10,3	2203	22,6	11,1	71	33,2	15,9	70	30,3	16,2
5	1818	21,0	10,6	2041	22,5	10,7	61	34,2	16,8	59	30,5	13,1
6	1760	21,1	11,1	1833	21,6	10,7	81	30,1	15,2	50	30,2	11,2
7	1729	20,4	10.8	1745	21,9	11,3	57	35,4	20,0	45	33,7	17,3
8	1810	20,5	11,1	1706	21,7	11,2	62	35,7	14,0	57	37,5	17,8
9	1794	19,6	11,1	1618	21,9	10,8	54	38,8	17,5	57	36,3	18,4
10	1804	18,7	10,5	1652	21,7	11,4	62	40,6	17,6	61	31,8	15,9
11	1936	18,0	10,7	1712	21,4	11,5	49	39,7	18,2	51	34,8	17,7
12	1881	17,4	11,1	1739	20,3	10,9	61	38,7	18,9	59	39,3	16,9
13	2054	16,7	11,5	1676	19,9	11,1	48	39,2	17,5	63	40,5	16,3
14	2224	16,4	12,0	1530	20,0	11,4	79	35,7	17,4	51	38,8	20,0
15	2439	16,1	12,1	1448	19,5	11,2	96	37,3	17,1	77	40,1	17,3
16	2492	16,5	12,4	1286	19,3	10,5	119	38,8	15,7	67	34,7	15,0
17	2468	16,6	12,8	1031	19,7	11,7	120	35,3	15,3	44	34,5	16,8
18	2190	17,1	13,6	755	20,8	12,8	84	37,9	15,4	49	38,0	17,8
Total	47,928	22,3	14,0	42,114	25,3	13,4	1798	32,7	16,9	1727	31,0	16,3

## Discussion

Turkey is located between 36–42° North, and 26–45° East. Between November and February, in higher latitudes the solar zenith angle is so oblique that few ultraviolet B photons reach the surface of Earth [15]. As low serum vitamin D levels are dependent on nutritional status and exposure to sunlight, findings reveal that as children grow up deficiencies become more apparent. This is one of the main reasons that seasonal variations should be recognized when evaluating vitamin D levels.

There have been a wide range of 25-OHD levels in studies from countries in the Middle Eastern region; possibly attributed to methodological and genetic variances [16–19]. The low levels of 25-OHD in our pediatric population were found in contrast to the reports of US NHANESIII study, where mean 25-OHD levels in over 2800 adolescents, aged 12–19 years, were between 26 and 36 ng/ml [15]. The inverse correlation between 25-OHD and iPTH levels have also been demonstrated previously in different studies [20–24]. In our study, the relationship between 25-OHD and iPTH was exponential and statistically significant (iPTH = 65.326 × 25-OHD^-0.28^, *r* = 0.347, *p* < 0.001). This finding is unique when compared to adults [25–29]. In our previous study we searched for the vitamin D cut-off level mainly in adults at 10 year intervals, while in this study we searched for vitamin D versus iPTH relationship at all pediatric ages [30]. Discussion over oscillations between 25-OHD and iPTH have also been published previosly and due to these studies there is still controversy over optimum 25-OHD level recommendations in the guidelines [31, 32]. Due to the fact 25-OHD levels show seasonal variances, determination of reference intervals can not be made accurately. Hill et al. analyzed the the relationship between serum 25-OHD and PTH in children and adolescents in the United States to determine the inflection point of 25-OHD for maximal suppression of PTH. This was in contrast to adults where the relationship between 25-OHD and PTH was linear with no inflection point [33]. In our study, using regression analysis, the 25-OHD level above which iPTH levels did not decrease was 25 ng/ml. In other studies values for this plateu vary from 16 to 60 ng/mL which may partially be explained by differences in ethnicity, age, gender, assays used and most importantly the method used to implement curve fitting and derive plateau [34–37]. Assessment of Vitamin D status can be assayed using radioimmunoassays, competetive protein binding assays (CPBA), high pressure liquid chromatography (HPLC), and liquid chromatography-tandem mass spectrometry. Because of an inter-assay variation these methods may yield different results; varying by up to 25% (at lower serum levels) and 10% with intra-assay. The optimal serum vitamin D concentration has not yet been established, and may change across different life stages. In our study 25-OHD levels were measured by a very sensitive method; LC MS being the most accurate method for 25-OHD level measurement. The cross sectional analysis of NHANES (National Health and Nutrition examination Survey) 2003–2006, which included 14,681 patient data older than 6 years, demonstrated that optimal 25-OHD level, defined as estimated maximum iPTH supression, does not occur until at least 25-OHD level is equal or higher than 40 ng/ml [38]. This is higher than our population based plateau. There were some limitations in our study, as it does lack body mass index, as well as other contents of fat as major determinants of 25-OHD. Secondly, as this study was a data-mining study, it could not unravel other predictors of low 25-OHD levels. These include polymorphisms of vitamin D metabolism pathways, other medical consequences, clothing habits, geographical location, weather conditions, skin color, hours of sun exposure, use of sun screen, use of calcium and vitamin D supplementation. Neverthless, our study does provide valuable insight on the grounds that it was based on retrospective data gleaned from tha largest data set in a pediatric population, in the literature to date. Due to the difficulty in maintaining adequate 25-OHD serum levels throughout the pediatric age while at the same time reacting to seasonal adjustments, more studies are needed to better understand the clinical implications of hypovitaminosis D and consequently, make recommendations for vitamin D supplementation.

## Conclusion

In conclusion our large data set demonstrated a high prevalence of hypovitaminosis D in Turkish children; most of whom reside in a sunny subtropical climate. This data is suggestive that a vitamin D level of 30 ng/mL would be a suitable criterion for 25-OHD deficiency in Turkish children. Population based cut-off levels for desired vitamin D levels must be identified in order to develop national recommendations of vitamin D supplementation.
